# Two Morphologies in a Single Placenta With Placental Mesenchymal Dysplasia

**DOI:** 10.7759/cureus.91177

**Published:** 2025-08-28

**Authors:** Snigdha Kumari, Sujata Siwatch, Minakshi Rohilla, Parikshaa Gupta

**Affiliations:** 1 Obstetrics and Gynaecology, Postgraduate Institute of Medical Education and Research, Chandigarh, IND; 2 Cytopathology and Gynaecologic Pathology, Postgraduate Institute of Medical Education and Research, Chandigarh, IND

**Keywords:** genetics, placental histopathology, placental mesenchymal dysplasia, placentomegaly, ultrasonography

## Abstract

An ultrasound investigation of a 28-year-old woman in the second trimester of her second pregnancy was suggestive of molar changes of placenta. Sonographically, the placenta had two distinct appearances - a normal portion and an area of heterogeneous appearance with a cystic area. It covered the internal os. There was a single live foetus. The management of this unique entity was marked by extensive workup of the differentials, testing and counselling. The pregnancy was continued, but antepartum haemorrhage resulted at 31 weeks, requiring an emergency cesarean delivery. Postpartum haemorrhage ensued, which required oxytocics and blood transfusion. Histopathology of the placenta was remarkable for features of placental mesenchymal dysplasia. The follow-up of the neonate and mother showed that they are doing well a year later. The diagnosis and management of this rare entity are discussed.

## Introduction

Placental pathology plays a critical role in the aetiology of adverse perinatal outcomes, including foetal growth restriction, preterm birth, and stillbirth. Abnormalities in placental structure and function are reported in a significant proportion of these cases, with studies suggesting placental lesions may be present in over 60% of unexplained stillbirths. These pathologies can range from vascular malformations and infarctions to more rare anomalies such as placental mesenchymal dysplasia (PMD). 

As Jauniaux et al. has highlighted, understanding the perinatal features associated with placental anomalies can enhance clinical decision-making and improve outcomes [1]. Accurate prenatal and postnatal recognition of such abnormalities is essential for diagnosis, prognosis and recurrence risk assessment. For example, PMD may mimic molar pregnancy on ultrasound but has a separate pathogenesis and clinical course [2]. Misinterpretation can lead to unnecessary interventions or missed opportunities for monitoring.

PMD has been reported to be associated with adverse perinatal outcomes, including foetal growth restriction, perinatal morbidity and mortality. We present a unique case with two different histologies in the placenta. The clinical case demonstrates a diagnostic dilemma and discusses the course of pregnancy of a woman diagnosed with an abnormal placenta on ultrasonogram.

## Case presentation

A 28-year-old gravida 2 para 1 was referred to our institute at 21 weeks of gestation with an ultrasound suggestive of molar changes in the placenta. Her previous pregnancy, three years back, had been uncomplicated, and she had undergone vaginal delivery at term. The index pregnancy was a spontaneous conception. There were no complaints of pain in the abdomen, bleeding per vaginum, hyperemesis, trauma or increased blood pressure record. The ultrasound with which the patient presented was remarkable for the presence of a normal posterior placenta in the upper segment, but with a large cystic mass 70*22 mm extending in the lower segment and covering the internal os, along with a single live intrauterine fetus corresponding to 20 weeks of gestation, with a doubt of molar pregnancy. Her vitals were stable with a pulse of 90/min and a blood pressure of 122/80 mmHg. The uterus was enlarged to 24 weeks of gestation size, and a single live intrauterine fetus corresponding to 20 weeks of gestation.

**Figure 1:**
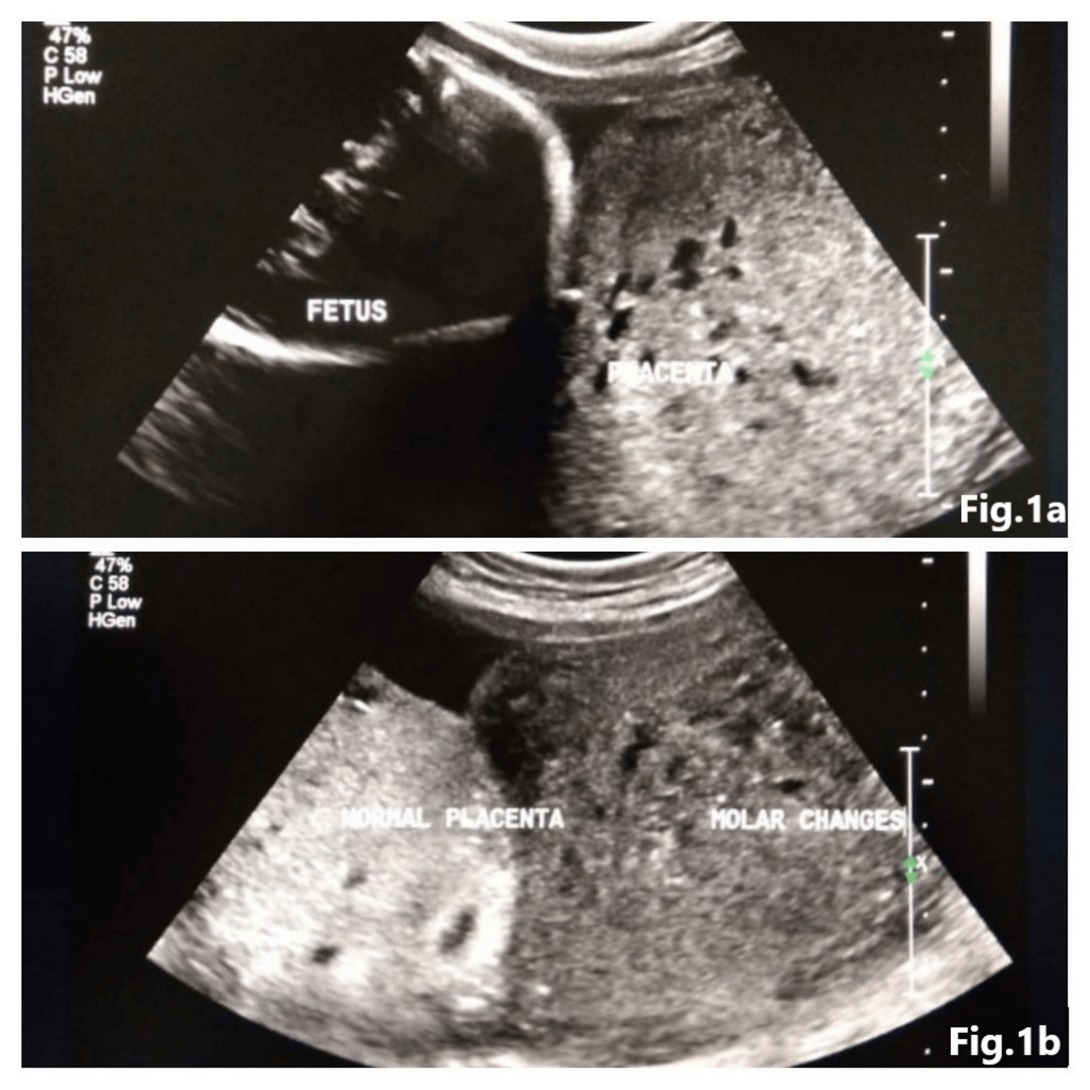
(a) Low-lying large placenta with anechoic spaces, which is also low-lying. (b) Two distinct morphologies in the placenta

Ultrasound showed placentomegaly (7.7 cm in thickness) with multiple anechoic cystic areas within the substance of the placenta, imparting the latter a Swiss cheese appearance alongside the sharply demarcated normal-looking placenta. (Figure 1). The placenta was posterior, starting from the fundus and reaching up to the os. Note was also made of a single live intrauterine foetus. The foetal anomaly scan was significant for only mild bilateral pyelectasis. Liquor volume was normal. Her beta-human chorionic gonadotropin (HCG) was >1,00,000 IU/mL. Her haemoglobin was 9.6 g/dL, total leucocytes 6500/ mL, platelets 2,30,000 /mL, thyroid-stimulating hormone (TSH) 0.005 mIU/ml and her blood group was B positive.

Considering the placentomegaly, our differential possibilities included molar gestation, partial mole, twin with single complete molar gestation, placental mesenchymal dysplasia and chorioangioma, among others. Amniocentesis and foetal karyotype were done in consultation with the patient. As the foetal anomaly scan and foetal karyotype were normal, she opted to continue the pregnancy. Patient was counselled regarding the diagnostic dilemma, anticipated complications with possible molar pregnancy that included risk of bleeding, early termination of pregnancy, risk of metastasis and the need for subsequent chemotherapy; and abnormal foetal karyotype in case of a partial mole. The higher risk of antepartum hemorrhage (APH), pregnancy-induced hypertension (PIH), preterm labour and foetal growth restriction (FGR) during the index pregnancy were discussed. She followed up in the outpatient department till at 31 weeks and two days of gestation, when significant proteinuria was first detected. However, her blood pressure remained normal throughout her pregnancy. Total proteinuria of 650 mg/24 hours was detected. Her beta HCG was raised (1,05,485.58 mIU/ml). Her hemogram and liver function test remained normal.

**Figure 2:**
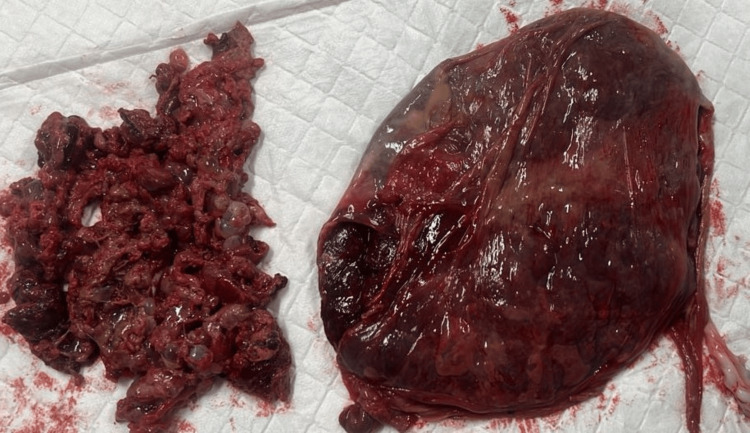
One part of the placenta was friable and demonstrated cystic vesicles ranging in size of 5-8 mm. Rest of the placenta was grossly within normal limits

The patient presented to the emergency department at 33 weeks and two days of gestation with profuse bleeding per vaginum. She was taken up for emergency caesarean section in view of symptomatic placenta previa. Intraoperatively, baby presented and delivered as breech with the Apgar score of 7.9 at one and five minutes and a weight of 1.74 kg. While part of the placenta with grape-like vesicles presented first followed by the normal-looking placenta (Figure 2). Multiple hemostatic sutures were taken to control bleeding from the placental bed and oxytocics to manage the bleeding. Total blood loss was estimated to be about one litre. One unit packed cells, random donor platelet and two fresh frozen plasma were transfused intraoperatively. Her post-operative stay was uneventful, and she was discharged on day 3. The placenta was remarkable for the presence of different morphologies and weighed 1700 g. 

**Figure 3:**
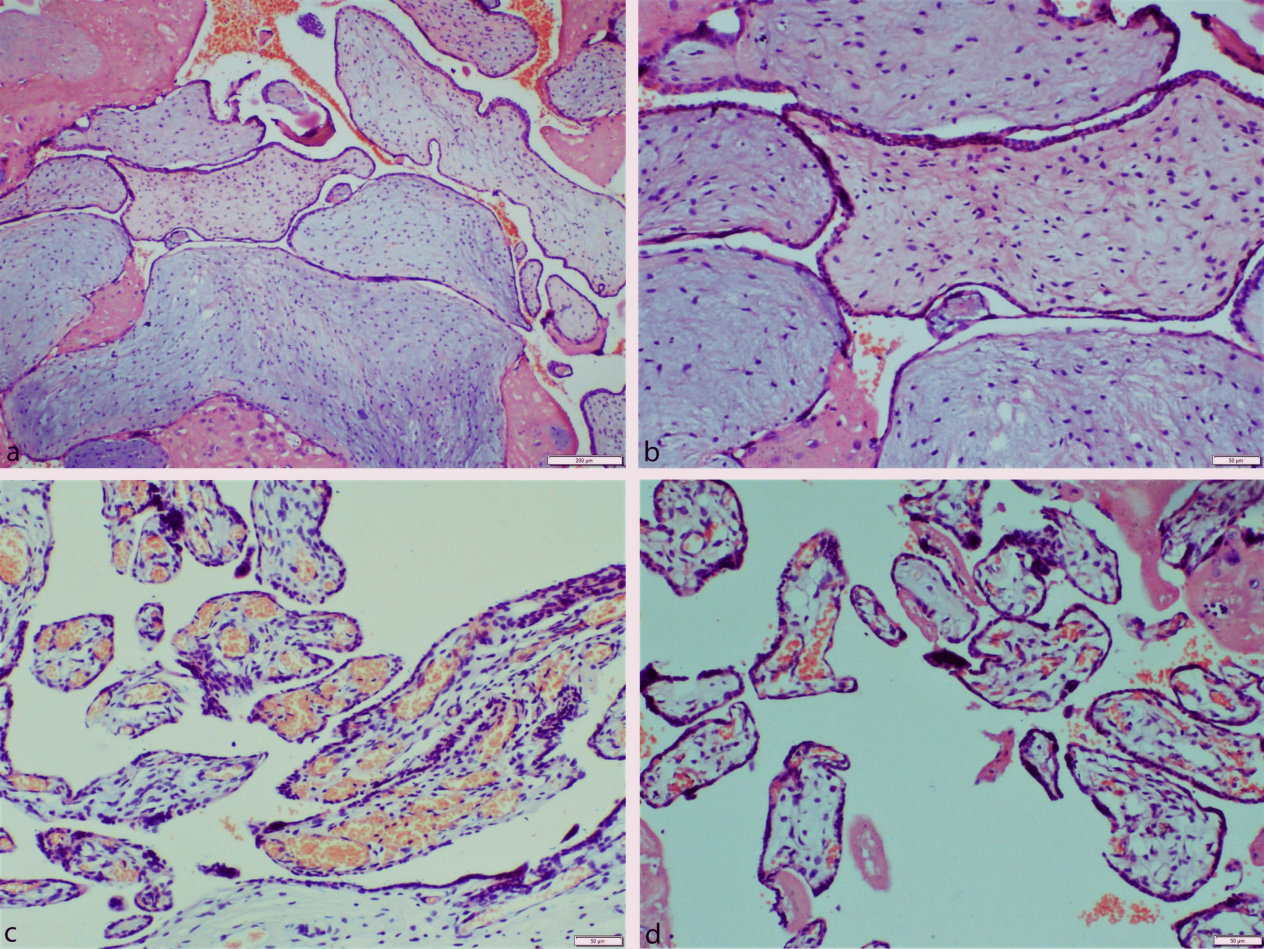
a, b: Sections from the cystic part of the placenta showing abnormally enlarged chorionic villi with myxoid degeneration without any trophoblastic proliferation (hematoxylin and eosin (H&E); a: 4x, b: 10x); c, d: Sections from the grossly normal part of the placenta showing normal third trimester chorionic villi without any evidence of myxoid degeneration and trophoblastic proliferation (H&E; 10x)

Gross examination of the placenta revealed two different parts (Figure 3). The larger part measured 24*13*8cm with highly coiled umbilical cord measuring 15 cm in length. Part of it was friable and had cystic vesicles of size 5-8 mm on the foetal surface. Normal part measured 13*8*5cm. Microscopic examination of the larger placenta showed scattered second and third trimester villi admixed with large hyalinized villi with hydropic changes and myxoid degeneration with focal areas of calcification (Figure 3). No trophoblastic proliferation was seen. Umbilical cord and membranes were unremarkable.

## Discussion

Placentomegaly can be seen in a significant number of pregnancies. Hydramnios, foetal hydrops and gestational diabetes are thought to be related to placentomegaly [1]. Apart from obstetrical associations in diabetes, rh-isoimmunised pregnancy and infections, placental pathology in itself may present like molar gestation - partial or twin gestation with complete mole in one sac, and placental mesenchymal dysplasia and tumors like chorioangioma. In the index case, the placenta was exceptional as it was large and had two very different appearances on ultrasound. There was no vascularity in the abnormal placental portion. Sonographic findings showed the placenta in a partial mole and a complete mole with co-twin look heteroechoic with partially solid and cystic areas and a chorioangioma mainly located on the foetal surface of the placenta with different echogenicity than the rest of the placenta [2,3]. It is important to distinguish PMD with partial mole because the latter may require pregnancy termination and from complete mole with co-twin since it is associated with the risk of persistent GTD, increasing maternal morbidity. We ruled out partial mole by doing a karyotype. Placental mesenchymal dysplasia was considered highly probable. Nonetheless, the patient was also counselled regarding the risk of developing foetal growth restriction (FGR), hypertension in pregnancy, antepartum hemorrhage, preterm labour and intrauterine foetal demise. FGR, foetal demise and maternal hypertension in PMD are implicated in changes in vascularity, shunting of foetal blood within vascular malformations, and thrombosis of blood vessels of stem villi, leading to hypoperfusion and hypoxia [4].

PMD is a fairly recently recognised, rare placental vascular anomaly. It was first described by Moscoso et al. in 1991 when they discovered two cases with enlarged placenta with features of partial mole on ultrasonogram and elevated maternal serum alpha fetoprotein [5]. Due to its unfamiliarity with the pathologists, PMD is both underdiagnosed and underreported with an incidence of 0.02% [6]. The etiology of PMD is currently not known, though some speculate it to be a congenital malformation of mesoderm [7]. It has 3.6:1 female:male predominance, which can be explained by androgenetic/biparental mosaicism [8]. PMD is suspected when sonographically we encounter a thickened placenta with hypoechoic areas resembling cystic spaces, as in our case. Apart from its association with foetal malformation, it can lead to compromised obstetrical outcome due to prematurity, abruption, growth restriction, hemorrhage and even stillbirths.

In nearly 25% of the cases, PMD is known to be associated with Beckwith-Wiedemann syndrome (BWS) characterised by macrosomia, exomphalos, macroglossia, omphalocele, craniofacial features and ear anomalies and internal visceromegaly, none of which were present on USG in our case [9]. Our patient developed proteinuria, but without hypertension, in the early third trimester. Our patient underwent caesarean section due to APH and delivered a phenotypically normal baby with average birth weight for that gestation, but a very large placenta with its weight equivalent to the weight of the baby. Postpartum hemorrhage resulted, needing oxytocics and transfusion. Neonatal hypoglycemia is also a frequent association with BWS, which can occur due to hyperinsulinemia secondary to islet cell hyperplasia of the pancreas [10]. Neonates of PMD who are phenotypically normal require follow-up for development of features of BWS and development of childhood tumours.

Placentas with placental mesenchymal dysplasia have been reported to weigh more than 90th in more than 90% of the cases [3]. Grossly, PMD placentas have dilated and tortuous chorionic plate vessels with multiple cystic vesicles similar to that of molar pregnancy [11]. Microscopically, these cystic vesicles correspond to dilated stem vessels with thickened vasculature surrounded by a mixture of normal-appearing and hydropic secondary and tertiary villi. These thick-walled vessels may show fibrinoid necrosis and degenerative changes in the vessel wall with varying degrees of luminal obliteration with thrombus [12,13]. Pathologically, the important distinguishing feature of PMD is the presence of dilated stem vessels and lack of trophoblastic proliferation [11].

Like neonates, maternal follow-up is also important to look for signs of persistent trophoblastic disease and recurrence of PMD in subsequent pregnancies, keeping in mind that 15% cases of BWS are familial. However, it is noteworthy that no signs of PMD has been found on a five-year follow-up [9].

## Conclusions

To conclude, placentomegaly is an important radiological finding which should be carefully evaluated as it has a bearing on foetal prognosis and preparedness for good obstetrical outcome. Placental mesenchymal dysplasia may be seen in a portion of the placenta and mimics a partial mole or twin gestation with mole in one sac. Histopathology of the placenta should be emphasised for women delivering large and abnormally looking placenta to differentiate it from molar gestation and plan maternal follow-up surveillance post-delivery.
